# Vaginal breech delivery: results of a prospective registration study

**DOI:** 10.1186/1471-2393-13-153

**Published:** 2013-07-24

**Authors:** Ingvild Vistad, Milada Cvancarova, Berit L Hustad, Tore Henriksen

**Affiliations:** 1 Department of Obstetrics and Gynaecology, Sorlandet Hospital HF, Kristiansand, Norway; 2 Department of Oncology, National Resource Center for Late Effects, Oslo University Hospital and University of Oslo, Oslo, Norway; 3 Women’s and Infant’s Division, Section of Obstetrics, Rikshospitalet, Oslo University Hospital and University of Oslo, Oslo, Norway

## Abstract

**Background:**

Most countries recommend planned cesarean section in breech deliveries, which is considered safer than vaginal delivery. As one of few countries in the western world Norway has continued to practice planned vaginal delivery in selected women. The aim of this study is to evaluate prospectively registered neonatal and maternal outcomes in term singleton breech deliveries in a Norwegian hospital during a ten years period. We aim to compare maternal and neonatal outcomes in term breech pregnancies subjected either to planned vaginal or elective cesarean section.

**Methods:**

A prospective registration study including 568 women with term breech deliveries (>37 weeks) consecutively registered at Sorlandet Hospital Kristiansand between 2001 and 2011. Fetal and maternal outcomes were compared according to delivery method; planned vaginal delivery versus planned cesarean section.

**Results:**

Of 568 women, elective cesarean section was planned in 279 (49%) cases and vaginal delivery was planned in 289 (51%) cases. Acute cesarean section was performed in 104 of the planned vaginal deliveries (36.3%). There were no neonatal deaths. Two cases of serious neonatal morbidity were reported in the planned vaginal group. One infant had seizures, brachial plexus injury, and cephalhematoma. The other infant had 5-minutes Apgar < 4. Twenty-nine in the planned vaginal group (10.0%) and eight in the planned cesarean section group (2.9%) (p < 0.001) were transferred to the neonatal intensive care unit. However, only one infant was admitted for ≥4 days. According to follow-up data (median six years) none of these infants had long-term sequelae. Regarding maternal morbidity, blood loss was the only variable that was significantly higher in the planned cesarean section group versus in the vaginal delivery group (p < 0.001).

**Conclusions:**

Strict guidelines were followed in all cases. There were no neonatal deaths. Two infants had serious neonatal morbidity in the planned vaginal group without long-term sequelae.

## Background

In 2000, the results of a randomized multicenter trial, the Term Breech Trial (TBT) were published in the Lancet [1]. 2083 women were included in the study, and of the 1,042 women assigned to planned vaginal delivery, 591 (56.7%) delivered by this method. The trial reported significantly lower perinatal mortality, neonatal mortality, or serious neonatal morbidity in the planned cesarean section arm (1.6%) versus in the planned vaginal delivery arm (5.0%).

The TBT has had a major impact on clinical practice, and most countries now recommend planned cesarean section in breech births [2]. Later, several methodological aspects of the study have been questioned [3-5]. Despite this, planned cesarean section is considered as the safest and therefore the most widely used delivery method according to several European publications [6-10]. As a response to the TBT, a Norwegian expert group in 2003 reviewed published international literature on breech births and obtained information on perinatal morbidity and mortality in term breech infants in Norway [11]. Their results showed a lower perinatal morbidity among infants born vaginally in breech presentation compared to both study groups of the TBT. The mortality rate was 0.31% when corrected for lethal malformations and 0.09% after the additional correction for death before admission to the maternity clinic [12]. This could be explained by the close fetal monitoring, national procedures, and sufficient skills of obstetric staff, combined with high quality neonatal service, in contrast to many of the participating clinics in the TBT. As a result, Norwegian delivery units have continued a practice with vaginal delivery provided that strict national guidelines are followed. As one of few countries in the western world which has continued to practice planned vaginal delivery in selected women, quality control of own practice is fundamental, and sharing current experience with vaginal breech deliveries has strongly been encouraged.

The purpose of this study is to evaluate prospectively registered neonatal and maternal outcomes in term singleton breech deliveries in a Norwegian hospital ten years after TBT. We aim to compare outcomes in term breech infants after planned vaginal delivery versus after elective cesarean section.

## Methods

### Data collection

Data on breech deliveries at our hospital has been prospectively collected in a comprehensive ‘breech database’ since 2001 to the present (variables listed below). Maternal delivery data as well as neonatal mortality and morbidity data are registered in the data base. Information on maternal complications was extracted from medical charts and the Partus database in retrospect. Medical charts were also reviewed for all neonates transferred to the neonatal intensive care unit (NICU) in order to verify and validate information. All women with a singleton, term gestation (>37 weeks) breech presentation between 2001 and 2011 were included. Exclusion criteria were preterm deliveries (< 37 weeks), multiple pregnancies, antepartum death and major congenital malformations.

### Mode of delivery

Breech deliveries were classified as planned vaginal breech deliveries if the woman delivered vaginally or by acute cesarean section after a previous decision of vaginal delivery, whether the decision was taken before labor or after a pelvimetry in early labor. Mode of planned delivery was recorded prospectively in the medical charts. Women with breech presentation were selected to vaginal delivery according to Norwegian guidelines (see below).

After spontaneous onset of labor, continuous electronic fetal monitoring was conducted in all cases with known breech presentation. In 2005, ST analyses of the fetal electrocardiogram (STAN) were introduced in breech deliveries. After spontaneous progress to umbilical level, the arms and the aftercoming head were actively delivered by the Lövsets and the Veit-Smellie-Moriceau maneuvers, respectively.

### Norwegian national guidelines for vaginal delivery of breech infants

1) Gestational age ≥ 34 weeks, 2) Estimated birth weight ≥ 2000 g and ≤4000 g (individual assessment between 4000–4500 g), 3) Pelvimetry (x-ray) was optionally performed in nulliparous women, in women with previous complicated vaginal birth, or previous birth of infant < 3000 g. Sagittal inlet diameter more than 11.0 – 11.5 cm and sum of pelvic outlet diameter more than 31.5 – 32.5 cm were accepted measures for vaginal delivery, 4) Frank breech or complete breech presentation, 5) No serious obstetrical complications or serious maternal diseases, 6) Adequate institution size (preferably more than 400–500 births a year), 7) Mode of delivery and delivery assisted by, or supervised by experienced gynecologists 8) Adequate anesthesia: epidural, pudendal nerve block, and prompt access to regional anesthesia if necessary.

### Neonatal outcome factors

Variables recorded in the breech database were: Apgar score <7 and <4 at 5 minutes, admission to NICU ≥ 4 days, brachial plexus injury, cephalhematoma, bone fracture, respiratory distress syndrome, mechanical ventilation treatment, continuous positive airway pressure (CPAP) treatment, and facial palsy. Gestational length was consistently based on ultrasound examination at 17–19 weeks, or if not available, the last menstrual period. Small for gestational age (SGA) was defined as having a birth weight lower than the 10th percentile calculated for the normal population based on data from Norwegian Birth Registry [13].

### Maternal outcome factors

Maternal age, parity (para 0, para ≥ 1), gestational length, birth weight, x-ray pelvimetry, and indication for mode of delivery were extracted from the breech database. Maternal complications were not originally registered, and were extracted from medical charts and the Partus database in retrospect. These variables include episiotomy, anal sphincter rupture, blood loss (dichotomized to ≤ 1500 or >1500 ml), need for blood transfusion, deep venous thrombosis, postoperative hematoma, wound infection requiring antibiotics, endomyometritis, and febrile illness > 1 day.

The primary outcome measure was neonatal mortality or serious perinatal morbidity similar to the criteria of the TBT [1] (Admission to NICU ≥ 4 days, cephalohematoma, bone fracture, respiratory distress syndrome, mechanical ventilation treatment, continuous positive airway pressure (CPAP) treatment, Apgar < 4 after 5 minutes).

### Statistics

Data were described with proportions for categorical variables and with median and ranges for continuous variables. Crude associations between categorical data were assessed with chi-square tests and t-tests for continuous variables. Non-parametric tests were applied when appropriate. The associations between categorical variables were quantified as odds ratios (ORs) with 95% confidence intervals (95% CI). P-values <0.05 were considered statistically significant. Data were analysed using the Statistical Package for Social Sciences (SPSS version 18.0).

### Ethics

The study was considered as quality assurance by the regional Committee for Ethics in Medical Research, Region South (2011/1006 D), and did not require approval or written consent. The Norwegian data inspectorate (2011/28688) approved the study.

## Results

**Figure 1:**
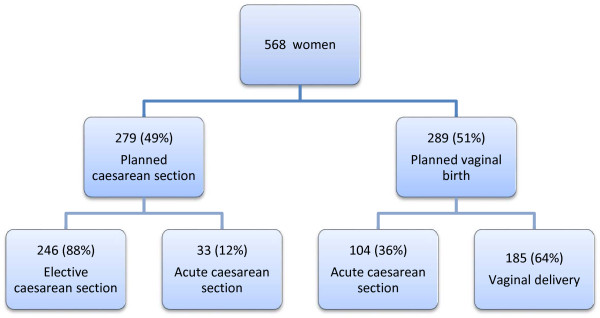
Study flow chart.

In the period from 01.01.2001 – to 31.12.2011, 571 of 16794 (3.4%) singleton term pregnancies were breech presentations at Sorlandet Hospital Kristiansand (Figure 1). Three cases were excluded due to known fetal anomaly. Of the remaining 568 women, elective caesarean section was planned in 279 (49%) cases, whereas vaginal delivery was planned in 289 (51%) cases. The guidelines were followed in all cases. We went through the medical charts for infants who were transferred to the NICU (37/568) after birth, with a median follow-up time of 6 years (range 1 – 11 years). One infant was diagnosed with Down’s syndrome post partum and one with autism at two years’ age. These two were not excluded from the analyses. Acute caesarean section was performed in 104 of the planned vaginal deliveries due to complications before or during labor. Hence, our study sample consisted of 185 vaginal births, which constituted 33% of the women with singleton fetuses in breech presentation at term. There were no neonatal deaths and two cases of serious neonatal morbidity in this period (outlined below).

Table 1 reports maternal and obstetric characteristics for the planned vaginal and caesarean section delivery groups. In Table 2, fetal outcomes for the two groups are reported. Thirteen percent (76/568) were SGA infants, with 51 infants in the planned vaginal group. Of these, a minority were transferred to the NICU (N = 11). Cord blood was sampled in 149 cases, 130 in the planned vaginal group and 19 in the planned cesarean section group. Acidemia (pH < 7.00) was reported in 5 (4%) cases in the planned vaginal group, and none in the planned cesarean section group. Two infants in the planned vaginal group had a cord blood base deficit of at least 15. Seven infants in the planned vaginal group had an Apgar score < 7 after five minutes versus none in the planned cesarean section group. Of these, one infant had an Apgar score < 4 after five minutes (serious morbidity). Thirty seven infants were transferred to the NICU, 29 in the planned vaginal group and eight in the planned cesarean section group (p < 0.001). Of these, one infant in the planned vaginal group was admitted for more than four days (serious morbidity). This infant was delivered vaginally, and had neurological morbidity comprising neonatal seizures, brachial plexus injury, cephalhematoma, and mechanical ventilation treatment. She is now seven years old, and according to the medical chart, she has been through several psychomotor tests showing ability levels within the normal range, although she demonstrates some mild visual-spatial impairment. The infant with Apgar score < 4 after five minutes was delivered with acute cesarean section due to fetal distress. Her Apgar score was 9 after 10 minutes, and she was discharged from NICU after one day’s observation. She is now twelve years old, and has shown normal cognitive and psychomotor development.

**Table 1:** Maternal and obstetric outcomes in the planned cesarean and planned vaginal delivery groups

	**Planned cesarean section (n = 279)**	**Planned vaginal birth (n = 289)**	**P-value**
Median (range) maternal age (years)	29 (19–42)	29 (16–43)	NS
Parity			
Nulliparous	154/279	152/289	
Multiparous	125/279	137/289	NS
Mean gestational age	38.7	39.4	
Mean birth weight (g)	3531	3399	<0.01
≥ 4000	50/279 (18%)	32/289 (11%)	
< 4000	229/279 (82%)	257/289 (89%)	0.02
Pelvimetry performed	111 (55%)	131 (60%)	0.18
Reasons for planned cesarean section	Fetopelvic disproportion 85 (35%)	0	
	≥ two cesarean sections 7 (3%)	0	
	Patients’ request 101 (41%)	0	
	Maternal disease 20 (8%)	0	
	Former obstetric complic. 5 (2%)	0	
	Other reasons 28 (11%)	0	
Reasons for acute cesarean section	Planned cesarean section 33 (24%)	Fetal malposition* 33 (24%)	
		Failure to progress 20 (15%)	
		Fetal distress 18 (13%)	
		Maternal disease 4 (3%)	
		Undiagnosed breech 11 (8%)	
		(patient’s request)	
		Other reasons 18 (13%)	

*Incomplete breech or footling presentation.

**Table 2:** Fetal outcomes in the planned cesarean and planned vaginal delivery groups

	**Planned cesarean section (n = 279)**	**Planned vaginal birth (n = 289)**	**P-value**
5-min Apgar < 7	0	7	<0.01
5-min Apgar < 4	0	1	NA
Transfer to NICU* < 4 days	8	29	<0.01
NICU ≥ 4 days	0	1	NA
SGA**	25	51	<0.01
SGA babies transferred to NICU	2	9	0.26
Fetal birth injury	0	1	NA
Umbilical artery pH < 7.00	0 (N = 19)	5 (N = 130)	NA

*Neonatal intensive care unit; **Small for gestational age.

In the planned vaginal delivery group, the number of successful vaginal deliveries was higher among multiparous versus nulliparous women (98/137 vs 87/152, p = 0.01). 82/568 (14%) infants had birth-weight ≥ 4000 g. Of these, vaginal delivery was planned in 40%, but only 15 infants were delivered by this method (weight range 4012 g – 4790 g). 2/15 had Apgar score < 7 after five minutes, but none were transferred to NICU. Fifteen percent (83/568) of the women had an undiagnosed breech presentation at birth, and of these 51 women (61%) delivered vaginally. Of those with vaginal delivery, an episiotomy was applied in 54%, and a third degree anal sphincter rupture was reported in 2% (Table 1). Of the maternal postoperative complications analyzed, mean blood loss was the only variable that was significantly higher in the cesarean section group versus in the vaginal delivery group (435 ml, SD 317 ml versus 359 ml, SD 231 ml) (p < 0.001). Four women had blood loss > 1500 ml in the planned vaginal group, compared to five in the planned cesarean section group. Table 3 reports management during labor and delivery for vaginal births.

**Table 3:** Management of labor and delivery in 185 vaginal births

	**N, %**
Induction of labor	3 (1.6)
Prostaglandins	2
Oxytocin	1
Augmentation of labor	148 (80.0)
Oxytocin	139 (75.1)
Amniotomy	9 (4.9)
Duration of labor (>4 cm dilatation)	
< 4 h	44 (24.6)
4–6 h	70 (39.1)
> 7 h	65 (36.3)
Duration of active pushing	
< 30 min	147 (81.7)
30–59 min	31 (17.2)
> 60 min	2 (1.1)
Station at beginning of active pushing	
High	12 (6.5)
Mid	47 (25.4)
Low	126 (68.1)
Pain-relief during labor	
Epidural	94 (50.8)
Fentanyl	43 (23.2)
Pudendal nerve block	8 (4.3)
Pethedin	4 (2.2)
STAN*	56 (30.3)

*ST Waveform Analysis initiated in 2003.

## Discussion

In our prospective single hospital based study, planned vaginal delivery of term breech infants was associated with low perinatal morbidity. There were no neonatal deaths among the breech babies in this period. Statistically significantly more infants in the planned vaginal delivery group had an Apgar score < 7 and were transferred to the NICU. However, only one infant was admitted for more than four days. Cord blood was only sampled in 19 of 279 planned cesarean section cases and 130 of 289 planned vaginal cases, and can therefore not be analyzed. According to follow-up data, none of the infants (including the infant with serious morbidity) have long-term sequelae due to mode of delivery.

Most authors conclude that cesarean section is associated with increased short term maternal morbidity. However, in the present study, we found no statistical significant differences between the groups except for higher blood loss in the surgery group, which was of no clinical significance. The long term effects of cesarean section on the risk of pregnancy and delivery complications are well documented [14-16]. These include placenta previa and accrete, preterm delivery and pre- and post partum bleeding, which may involve a risk for both mother and child.

Most studies of term breech deliveries are retrospective and based on registry data, which make comparisons difficult because of lack of antenatal and postnatal information. Our study is in terms of design comparable to a large prospective observation study from France/Belgium that included 8105 women [17], According to that study, vaginal delivery of breech infants remains standard practice in France. The proportion of planned vaginal deliveries was 51% in 1998 and decreased to 31% in the study period (2001–2002). In our study, vaginal delivery was planned in 51% of breech births throughout the study period (varying from 45% in 2003 to 57% in 2010). In the study by Goffinet et al. [17], 54 (2%) in the planned vaginal group were transferred to the NICU, versus (29) 10% in our study. However, in our study only one of 29 infants was admitted at the NICU for more than 4 days, compared to 23/54 (43%) of the infants in the French/Belgian study. The main reason is probably variations in routines, including a low threshold for transfer to the NICU at our hospital, reflected by the short admission time. Cultural and traditional differences between the countries may also explain variations in management of delivery. In the French/Belgium study, duration of labor (first stage) was ≥ 7 hours in 1.4% versus 36.3% in our study. Norwegian doctors and women seem to accept longer duration of labor before cesarean section is decided. In our study active pushing started after the presenting part had reached the outlet in 93.5%, in accordance with the Goffinet study (96.4%). Furthermore, the percentage of women with active phase of the second stage of labor (i.e. pushing) longer than 60 minutes was only 2/185 (1.1%), in line with the French/Belgian study (0.2%). According to the Norwegian guidelines, active pushing should be awaited until the presenting part reaches the outlet and is thus followed in a majority of the deliveries. In contrast, active phase of second stage of labor longer than 60 minutes was 5.0% in TBT possibly due to earlier start of pushing than in our study. Shorter duration of active pushing is known to increase the chances of a vaginal birth [18].

In contrary to the findings of the TBT and several retrospective studies [1,6-9], we found no excess risk for neonatal mortality or serious morbidity in the planned vaginal delivery group versus in the planned cesarean section group. As the rate of vaginally delivered breech infants is much higher at our institution (33%) compared to other studies, comparisons are difficult to make. Today approximately 20% of breeches are delivered vaginally in the Netherlands compared to 50% before the TBT [7]. The numbers are even lower in Scotland, Ireland and Denmark; 5 – 10% today versus 20 – 23% before the TBT [10,19,20].

There is a risk that reduction in vaginal breech deliveries may lead to less skilled obstetricians and less favorable results in situations where vaginal delivery is unavoidable, such as undiagnosed breech in advanced labor or delivery of second twin. In the prospective French/Belgian study, 32% of the breech deliveries went vaginally and the study included only one neonatal death of a non-malformed infant (in the cesarean section group) among 8105 term breech deliveries [17]. Furthermore, except for a 5-minute Apgar score less than 4 in a total of five infants, no difference in severe outcomes was observed between the two groups. Also, the authors of a Finnish study conclude that selectively vaginal breech deliveries may be safe in hospitals where the obstetricians have a tradition of vaginal deliveries [21]. In their study (years 1995 – 2002), 455 of 986 (46%) term breech infants were delivered vaginally. When infants with congenital malformations were excluded, they reported no perinatal deaths in their material. This is in line with the conclusions of an Austrian observation study comprising 211 breech presentation pregnancies where 46 of 85 planned vaginal deliveries actually went vaginally [22]. Common features of the French/Belgium, the Finnish, the Austrian and our study, include strict selection criteria for whom should be offered vaginal delivery, close fetal monitoring and the presence of skilled obstetricians. The relatively high frequency (15%) of undiagnosed breech presentation at birth in our study has also been found in other studies (17-21%) supporting the importance of being able to handle an unexpected breech delivery vaginally [23-25].

The present study is not sufficiently powered to demonstrate differences in perinatal morbidity or mortality between the groups, as these outcomes are rare. In a Norwegian paper on breech deliveries, sample size calculation was done based on an anticipated perinatal mortality of 0.25% in the planned vaginal group and 0.1% in the cesarean group [12]. The authors concluded that a Nordic study similar to the TBT would require a total number of 10,000 to be recruited in each group for two-sided testing, a number exceeding practical limits [12]. Randomized controlled trials are considered as the gold standard for clinical trials, but the appropriateness of this design for studies of complex clinical procedures like breech deliveries has been questioned [5,26]. A strength of the present study is the comprehensive prospective registration conducted by midwifes at our institution. Furthermore, we were able to review the hospital records of all the infants that were admitted to the NICU after birth. In this study, the groups were compared according to planned mode of delivery before onset of birth. However, we have no information on fetuses turned to cephalic before delivery. We cannot control for all confounding factors, including lack of information about education level and maternal pregestational morbidity. Further, we can also assume that there are psychological differences between the groups, as maternal request was the reason for planned cesarean section in 41% of the cases of planned cesarean section. However, antenatal care is uniform in Norway, and more than 90% of pregnant women are followed up by their general practitioner and/or community midwife [27]. Furthermore, pre-labor, all women with known breech presentation are counseled by experienced gynecologists and selected to planned vaginal delivery after a thorough assessment.

## Conclusions

Our findings reflect a well-functioning health care system where vaginal delivery of breech babies has been practiced even after the results of the TBT was published. The present study does not have enough power to make a definite conclusion that vaginal breech delivery is completely safe. Yet, our results indicate that vaginal delivery of term breech infants is acceptable provided the following conditions: Selection guidelines are followed, the fetal monitoring is of high quality, and the volume of breeches delivered vaginally is sufficient to maintain a high level of competence among obstetricians. The women should also be counseled about the increased risk of short-term NICU admission.

## Abbreviations

TBT: Term Breech Trial; NICU: Intensive care unit; SGA: Small for gestational age; CPAP: Continuous positive airway pressure; STAN: ST analyses of the fetal electrocardiogram; ORs: Odds ratios; CI: Confidence intervals; SPSS: Statistical Package for Social Sciences.

## Competing interests

There are no conflicts of interest for any of the authors. There are no financial competing interests.

## Authors’ contributions

IV contributed in acquisition, analysis and interpretation of data. She drafted and revised the manuscript. MC contributed in analysis and interpretation of data and revised the manuscript. BLH contributed in acquisition of data and revision of the manuscript. TH contributed in interpretation of data and revision of the manuscript. The four authors approved the final version of the manuscript.

## Pre-publication history

The pre-publication history for this paper can be accessed here:

http://www.biomedcentral.com/1471-2393/13/153/prepub
